# Spontaneous pneumomediastinum after bench press training

**DOI:** 10.1002/ccr3.857

**Published:** 2017-02-23

**Authors:** Tomoya Nishino

**Affiliations:** ^1^ Department of Emergency and Critical Care MedicineTokai University School of MedicineShimokasuya143Isehara cityKanagawa259‐1193Japan

**Keywords:** Bench press training, chest pain, pharyngeal pain, pneumomediastinum

## Abstract

Spontaneous pneumomediastinum is often associated with asthma and mainly affects adolescent males with a tall, thin body habitus. A 17‐year‐old man complained of chest and pharyngeal pain after bench press training and spontaneous pneumomediastinum was diagnosed. It should be considered in the differential diagnosis of chest pain of uncertain cause.

Question: What is an important consideration in chest pain after training?

Answer: pneumomediastinum.

A 17‐year‐old man complained of chest and pharyngeal pain after bench press training. Although pneumothorax was not observed, pneumomediastinum was detected using plain chest computed tomography (CT) (Fig. 1). Upper gastrointestinal examination and fiber‐optic bronchoscopy were performed. Upper gastrointestinal examination revealed no leakage of contrast agent, and fiber‐optic bronchoscopy revealed absence of hematoma in the tracheal lumen (Fig. 2). Spontaneous pneumomediastinum was diagnosed, and the patient was treated by making him nil by mouth and initiating antibiotics. Pneumomediastinum was resolved on day 3, and he was discharged. Spontaneous pneumomediastinum should be considered in patients with chest pain after exercise.

**Figure 1:**
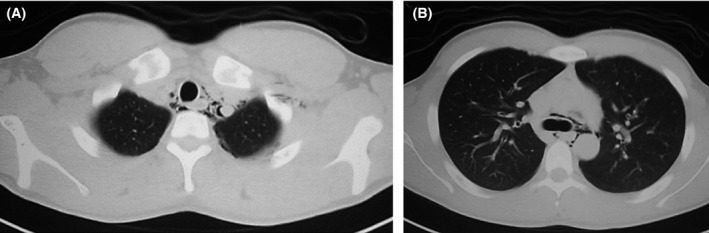
plain chest computed tomography showing pneumomediastinum.

**Figure 2:**
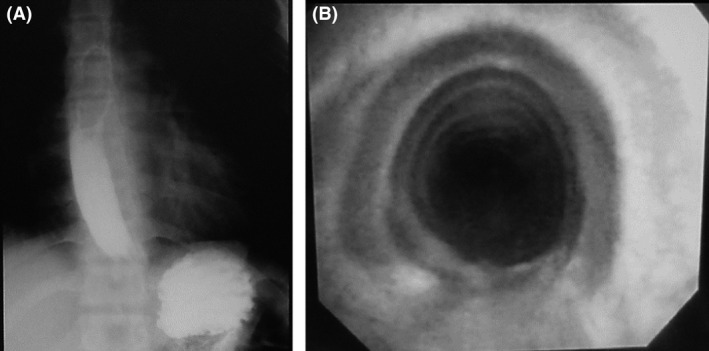
Upper gastrointestinal examination (A) showing no leakage of contrast agent and fiberoptic bronchoscopy (B) showing absense of hematoma in the tracheal lumen.

## Conflict of Interest

The authors state that they have no conflict of interest.

## Authorship

TN: contributed to conception and design of the article, acquisition of data, analysis and interpretation of data, drafting the article, revising it critically for important intellectual content, and final approval of the version to be published.

